# 

**Published:** 1882-02

**Authors:** 


### CONTENTS.

COMMUNICATIONS.

A General Treatment of Irregularities........ 24

SELECTIONS.

Development of the Enamel........ 62
Some Hints on the Preparation and Mounting of Microscopic Objects........ 68
Sanitary Condition and Longevity of Jews........ 81
Female Students at the Pennsylvania Hospital........ 82
Development of the Eyes........ 83
A Singular Suicidal Attempt........ 84
General Acceptance of Antiseptic Methods........ 85
Aphthon's Sore Mouth of Infants........ 85
On the Cause of Death Under Chloroform........ 86
To Prevent Plaster from Adhering to Rubber Plates in Vulcanizing 87
Sir Erastmus Wilson........ 88
How Printing Affects the Health........ 88
The Effects of Ozone........ 89
The General and Special Education of Dentists........ 90

EDITORIAL.

The Annual Meeting of the Oldest Dental Society........ 99
A Good Bracket........ 100
A New Journal........ 100
The Ohio State Journal of Dental Science........ 101
Obituary........ 101
Gone Hence........ 102

CORRESPONDENCE.

The Dentist of Bigler's Gulch........ 77
The Value of Mental Tension........ 80

